# Multidimensional background examination of young underweight Japanese women: focusing on their dieting experiences

**DOI:** 10.3389/fpubh.2023.1130252

**Published:** 2023-06-02

**Authors:** Yuka Murofushi, Shinji Yamaguchi, Haruka Kadoya, Hikaru Otsuka, Kasane Ogura, Hideyoshi Kaga, Yasuyo Yoshizawa, Yoshifumi Tamura

**Affiliations:** ^1^Faculty of Health and Sports Science, Juntendo University, Chiba, Japan; ^2^Graduate School of Health and Sports Science, Juntendo University, Chiba, Japan; ^3^Division of Public Health, Department of Hygiene and Public Health, School of Medicine, Tokyo Women’s Medical University, Tokyo, Japan; ^4^Faculty of Medical Science, Juntendo University, Chiba, Japan; ^5^Department of Sports Medicine and Sportology, Juntendo University Graduate School of Medicine, Tokyo, Japan; ^6^Sportology Center, Juntendo University Graduate School of Medicine, Tokyo, Japan; ^7^Juntendo Advanced Research Institute for Health Science, Juntendo University, Tokyo, Japan; ^8^Department of Metabolism and Endocrinology, Juntendo University Graduate School of Medicine, Tokyo, Japan; ^9^Center for Healthy Life Expectancy, Juntendo University Graduate School of Medicine, Tokyo, Japan; ^10^Faculty of International Liberal Arts, Juntendo University, Tokyo, Japan

**Keywords:** underweight, BMI, birth weight, dieting experience, exercise habit, eating habit, psychological effects

## Abstract

**Introduction:**

This study examines the background of underweight young women in Japan from multiple perspectives, focusing on whether they have ever dieted.

**Methods:**

A screening survey was administered to 5,905 underweight (BMI < 18.5 kg/m2) women aged 18–29 years, who could report their birth weight recorded in their mother-child handbook. Valid responses were obtained from 400 underweight and 189 normal-weight women. The survey collected data regarding height, weight (BMI), body image and perception of weight, dieting experience, exercise habits from elementary school age onwards, and current eating habits. Additionally, five standardized questionnaires were used (EAT-26, eHEALTH, SATAQ-3 JS, TIPI-J, and RSES). The primary analysis was a comparative analysis (t-test/χ2)—with the presence or absence of underweight and diet experience as independent variables, and each questionnaire as a dependent variable.

**Results:**

The screening survey revealed that approximately 24% of the total population was underweight, with a low mean BMI. Of the respondents, more than half reported their body image as skinny and a small percentage as obese. Compared with the non-diet-experienced group (NDG), the diet-experienced group (DG) had a significantly higher proportion of past to current exercise habits. There was a significantly higher percentage of disagreement responses from the DG for weight and food gain than for the NDG. The NDG weighed significantly less than the DG in terms of birth weight, and lost weight easier than the DG. Additionally, the NDG was significantly more likely to agree with increasing weight and food intake. The NDG’s exercise habits were below 40% from elementary school age to the present, predominantly owing to a dislike for exercise and a lack of opportunity to implement it. In the standardized questionnaire, the DG was significantly higher for EAT-26, eHEALTH, SATAQ-3 JS, and Conscientiousness (TIPI-J), whereas the NDG was only significantly higher for Openness (TIPI-J).

**Discussion:**

The results suggest the need for different health education programs for underweight women who desire to lose weight and experience dieting and for those who do not. This study’s results are reflected in the development of sports opportunities optimized for each individual, and in the development of measures to ensure adequate nutritional intake.

## 1. Introduction

What characteristics and backgrounds exist among young, underweight women that influence their thinness? Recent studies have shown that young women who are thin, eat little, and are inactive have a high risk of diabetes, similar to obese individuals (1). Accordingly, health hazards associated with thinness have attracted increasing attention. Based on body mass index (BMI) measures, underweight (BMI < 18.5 kg/m^2^) and severe obesity (BMI > 35 kg/m^2^) are associated with a significantly higher risk of adverse health effects (2). As obesity is often caused by lifestyle habits, such as overeating and lack of exercise, international research trends are particularly active worldwide regarding this topic. Severe obesity is reported in 6% (approximately 1 in 20) of men and 9% (approximately 1 in 10) of women worldwide (3). However, being underweight remains prevalent in the world’s poorest regions, especially South Asia (3). Even in Japan, a developed country, the situation is grave, with approximately 20% of young women in their teens and 20s being underweight, with a BMI of less than 18.5 kg/m^2^. According to a Japanese Ministry of Health, Labor and Welfare, a survey, the average BMI of women, aged 15–24, decreased from 21.5 in 1960 to 20.5 in 1995 (4). The National Health and Nutrition Examination Survey report also reported that the percentage of underweight women in the 20–29 age group reached 29.0% in 2010, but remained high at 20.7% in 2019 (5). Consequently, examining the background of young women’s thinness and studying its relationship to future health hazards and preventive measures is an urgent task.

Underweight women are more likely to exhibit poorer psychological health than normal-weight women (2). In particular, for adolescents, a gap between body image and actual BMI is evident. Even among thin individuals, dissatisfaction with body shape is high, and a positive correlation has been found between mental health, self-esteem, and body dissatisfaction (6). Thus, those who are dissatisfied with their bodies exhibit lower self-esteem. An association between body dissatisfaction, high stress levels, and low self-esteem has also been found (7). Furthermore, a low dietary intake and lack of exercise are also characterized by thinness among young women (1), which should be eliminated owing to the misconception that thinness is healthy. However, the following two types of trends were noted: those who lost weight without dieting and those who lost weight after dieting. If these differences in background are considered, the approach to health awareness for underweight women must be revised. Underweight women who desire to lose weight and who do not exhibit different eating habits, and women who desire to lose weight, tend to pay particular attention to improving their perception of their body constitution and eating habits (8). Moreover, underweight women have been reported to use their weight and shape to appraise their body image (9). A survey of college-age women revealed that those with high skeletal muscle mass perceived their bodies as obese and exhibited a greater desire to lose weight and undertake a dieting experience, than those with low skeletal muscle mass. Moreover, reportedly, those with low skeletal muscle mass exhibit a higher percentage of childhood dislike for exercise (10). These factors may also influence physique, body shape, body image, and other factors that affect thoughts regarding thinness, dieting behavior to lose weight, or exercise habits. However, these conditions must be thoroughly examined. For example, the experience of dieting to lose weight may also differ in terms of thoughts regarding ideal body weight and shape, eating habits, eating behavior, perception of food (eating attitude) (11), and exercise habits from the past to the present. Additionally, women who desire to lose weight are more likely to internalize information from the media (12), suggesting that underweight women who have dieted may be more susceptible to these influences. Furthermore, e-health literacy (13)—the skill of using information technology for health—suggests that those who have experienced dieting are more likely to actively seek information to ensure it is effective. Therefore, e-health literacy is expected to increase.

Moreover, weight perception and self-esteem are related (14), and weight and shape dissatisfaction has been demonstrated to impact negative psychological functioning, such as depression, especially concerning self-esteem (15). Self-esteem is considered a subjective indicator of acceptance or rejection of a person’s current self by others (16). This suggests that underweight women may be more conscious of their body image and are more concerned regarding how others perceive them. It is yet to be clarified how personality traits, such as the Big Five personality traits (17), differ depending on the dieting experience of underweight women. As the direction of health promotion education is diverse, examining the background of underweight young women from multiple perspectives—to determine the direction of future health education and awareness-raising activities—is necessary.

Therefore, this study examines the multifaceted effects of diet experience on weight perceptions, past and present exercise habits, eating behavior and eating habits, media influence on body image, e-health literacy, personality traits, and self-esteem among young underweight Japanese women. For birth weight, the reported data were compared using Maternal and Child Health (MCH) Handbooks. We examined exercise habits from childhood to adolescence, to determine when inactivity was formed. Based on these data, examining sports opportunities and nutritional intake optimized for each individual will contribute to women leading long, healthy, and prosperous lives.

## 2. Methods

### 2.1. Participants

**Figure 1:**
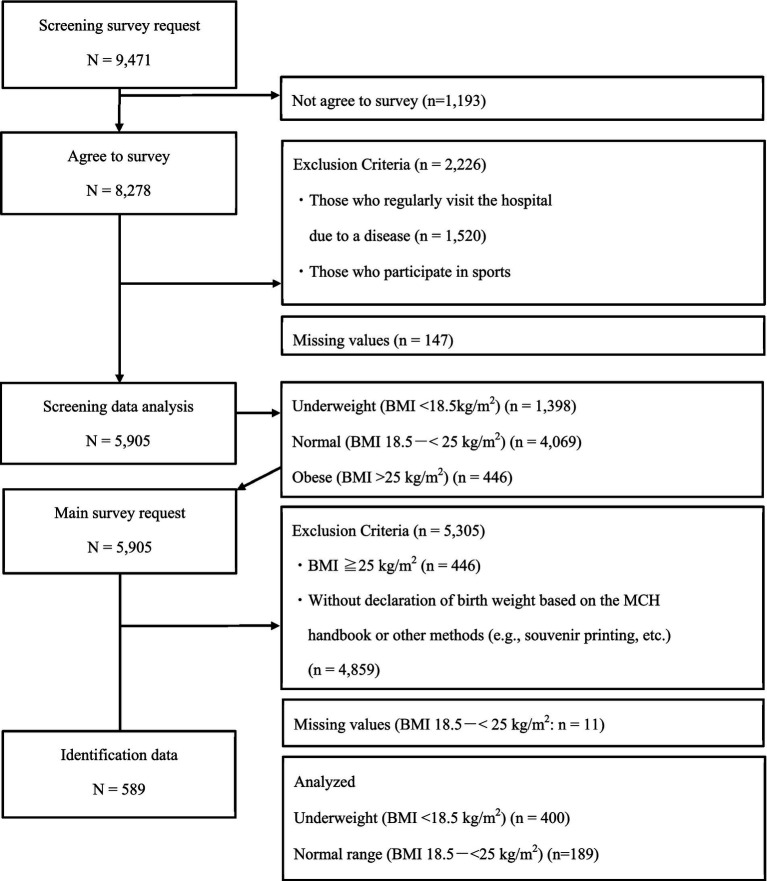
Flowchart of participant extraction. *Note*. BMI: body mass index. MCH: Maternal and Child Health.

This study was conducted as a feasibility study. It included women aged 18–29 years from Japan. We recruited 200 underweight women (BMI <18.5 kg/m^2^) with and without previous dieting experience and 200 normal-weight women (BMI 18.5–25 kg/m^2^). Furthermore, we recruited participants who could accurately tell their birth weight. Therefore, the inclusion criteria were those who could report their birth weight, as recorded in their MCH Handbook. The exclusion criteria were current hospitalization for any disease and sports activity requiring weight loss. Figure 1 depicts a flowchart of the study’s target population. First, a screening survey was conducted to identify participants who fit the above criteria; 9,471 participants indicated that they would consent to participating in the survey. Of these, 5,905 valid responses were obtained. This survey’s results included underweight (BMI <18.5), normal-weight (BMI 18.5 - <25), and obese (BMI >25) participants. Next, survey respondents who were screened as underweight and normal weight were included in the primary survey. The final number of valid responses from respondents who agreed to participate in this survey was 400 underweight and 189 normal-bodied women. In the underweight group, 304 participants reported birth weight based on the MCH Handbook.

### 2.2. Survey methodology, period, and ethical considerations

The survey method used in this study involved recruiting registered respondents from a Japanese internet research company. The survey period was May to June 2022. A survey request was sent to women who were registered as monitors with to company. Response data was collected for the screening and primary surveys, each with a four-day response period. For the primary survey, regardless of the recruitment period, the survey did not accept responses once it reached its required number of recruited participants. From the descriptive statistics of the Japanese National Health and Nutrition Survey, the research company considered that approximately 30% of women aged 15–19 years and 20% of women aged 20–29 years had a BMI less than 18.5 (4, 5). The Japanese national monitors registered with the survey firm had approximately 2,000 underweight young women. Based on the survey company’s previous experience, a 20–30% decrease in attrition rate was predicted when moving from screening to primary surveys. Moreover, the number of responses was lower than estimated due to the condition of those who could report their birth weight based on their MCH Handbooks. For both underweight and normal-weight individuals, approximately one-third of each population in the screening survey reported their birth weight according to the MCH Handbook. As for potential bias, the research company’s previous collection trends were expected to indicate those with many respondents in the 22 or 23+ age groups.

This study was conducted after review and approval by the research ethics committee of the first author’s institution (IRB number: 2022–39). The survey description page included an overview of the study and response times (approximately 25–40 min). The participants who consented to participate in the study were asked to complete the survey. The description page also explained that personal privacy would not be violated, that the data set delivered by the survey company would be kept anonymous, and that it was practically impossible to exclude individuals after collection, as they could not be identified. The participants could withdraw consent to the study and discontinue their participation at any time of their own free will and without any disadvantage.

### 2.3. Survey items

The survey in this study was classified into the following two phases: a screening survey and primary survey. Supplementary Table S1 presents the survey items and questionnaire for each phase. The questions asked in the primary survey are denoted by [M] (= Main survey question) in this table. The survey items were developed based on the areas of expertise of the first author (Sports Medicine and Sports Psychology) and coauthors (Athletic Training and Kinesiology, Healthy Life Expectancy, Health Psychology, Metabolism and Endocrinology, Sports Medicine and Sportology), and their surface and logical validity were examined.

First, for (a) *Demographic data,* we asked questions regarding current age, first menarche age, current height and weight, as well as birth weight recorded in MCH Handbook, highest weight ever recorded, ideal weight, unacceptable weight, weight perceived as a model’s shape, and obese weight. We also examined the annual income of our participants to ascertain their economic disparities. According to a 2022 survey by Japan’s National Tax Agency, the average *per capita* salary for salaried workers who worked throughout the year was 4,430,000 yen; for women, this was 3,020,000 yen (18). Next, for (b) *Diet experience*, we asked regarding body image, weight fluctuation, gaining weight, (c) *Exercise habits*, (c-1) *Reasons for exercise habits*, (c-2) *Reasons for not having exercise habits,* and (d) *Eating habits*. Exercise habits were defined as “exercising at least twice a week for at least 30 min each time for at least one year.” Participants who answered “Yes” to (c) *Exercise habits* were asked to answer c-1. *Reasons for exercise habits* and to indicate the number of days and hours per week they exercise. Those who answered “no” to (c) *Exercise habits* answered c-2. *Reasons for not having exercise habits.*

A standardized questionnaire was used in this study (Table 1). The questionnaires were: (1) EAT-26 (11, 21), (2) eHEALS (13, 22), (3) SATAQ-3 JS (12, 23), (4) TIPI-J (17, 24), and (5) RSES (20, 25). The Japanese version was used for all participants.

**Table 1:** Standardized questionnaire list (conducted in the primary survey).

No	Scale	
1	Eating Attitude Test (EAT-26, Japanese version)	
	EAT-26 is 26 items that measures eating attitude and comprises five factors: Dieting, bulimia, food preoccupation, and oral control related to self-control about eating and perceived pressure from others to gain weight.	•EAT-26 contains 26 items, scored on a 4-point Likert scale (0–3), with a possible total of 0–78 points. Responses are Always (3), Usually (2), Often (1); and Sometimes (0), Rarely (0), and Never (0), and a final score is calculated by summing 26 items. •EAT-26 score 9 or less: normal, 10–19: midrange disordered eating attitudes, 20 and above: disordered eating attitudes (65).
2	Japanese version of eHealth Literacy Scale (eHEALS)	
	The eHEALS is an 8-item scale and consists of one factor scale that measures perceived skills at finding, evaluating, and applying electronic health information to health problems.	•eHEALS contains 8 items, measured with a 5-point Likert scale, with response options ranging from strongly disagree (1) to strongly agree (5). •Total scores of the eHEALS are summed to range from 8 to 40, with higher scores representing higher self-perceived eHealth literacy (13). •The reliability of the SATAQ −3 JS was Cronbach’s alpha coefficient of 0.93. •The scale mean score was 23.5 ± 6.5 points.
3	Sociocultural Attitudes Toward Appearance Questionnaire-3 Japanese Short Version (SATAQ-3 JS)	
	The SATAQ-3 JS is 12-item scale that measures endorsement of sociocultural beauty standards depicted in the media. The SATAQ-3 has four factors/subscales: Information (INFO), Perceived Pressure (PRESS), Internalization-General (INT-GEN), and Internalization-Athlete (INT-ATH).	•Items are rated on a 5-point Likert scale ranging from definitely disagree (1) to definitely agree (5). Total score of SATAQ-3 is summed to range 12 to 60, with higher scores indicating greater media endorsement. •The reliability of the SATAQ −3 JS was Cronbach’s alpha coefficient of 0.89. •The scale mean was 39.58 ± 9.76 points, and the median was 40 points. 1.8% of the respondents gave a perfect score of 60 points.
4	Japanese version Ten-Item Personality Inventory (TIPI-J)	
	The TIPI-J is 10-item scale that measures each of the five factors of the Big Five. The TIPI-J has five factors/subscales: Extraversion, Agreeableness, Conscientiousness, Neuroticism, and Openness.	•Items are rated on a 7-point Likert scale ranging from disagree strongly (1) to agree strongly (7). •The reliability of the TIPI-J (Cronbach’s alpha coefficient) was as follows: Extraversion at 0.78, Agreeableness at 0.77, Conscientiousness at 0.77, Neuroticism at 0.84, and Openness at 0.76. •The scoring method is as follows; Extraversion: item 1 + (8-item 6). Agreeableness: (8-item 2) + item 7, Conscientiousness: item 3 + (8-item 8), Neuroticism: item 4 + (8-item 9), Openness: item 5 + (8-item 10). •Higher scores reflect personality tendencies for each factor. •The means and standard deviations at the TIPI-J development were as follows; Extraversion: 7.83 ± 2.97, Agreeableness: 9.48 ± 2.16, Conscientiousness: 6.14 ± 2.41, Neuroticism: 9.21 ± 2.48, Openness: 8.03 ± 2.48.
5	Rosenberg Self-Esteem Scale (RSES Japanese version)	
	The RSES is devised by Rosenberg, and consists of 10-items and one factor scale, that quantifies global positive and negative attitudes toward the self.	•RSES Japanese version contains 10 items, measured with five responses in a Likert scale including intermediate items: strongly agree, agree, neither disagree, and strongly disagree. •Negative items (reversal items) need to be converted to 5 points ↔ 1 point, 4 points ↔ 2 points, and then added (3 points were left unchanged). •Total scores of the RSES Japanese version are summed, to range from 10 to 50. •The higher the score, the higher the level of self-esteem. •The Japanese version, created by (20), is a 5-point Likert scale that includes an intermediate item (neither), as a choice. The original version uses a 4-point Likert scale, but since the validity and reliability of the Japanese version has been examined using the 5-point Likert scale, we decided to use this version for the survey.

### 2.4. Analytical methods

#### 2.4.1. Screening survey

The screening survey was analyzed for demographic data, exercise habits, and eating habits based on the characteristics of underweight, normal weight, and obesity. First, we calculated the mean and standard deviation of (a) *demographic data*. BMI values were calculated using the participants’ reported height and weight data for the weight variables. Data on birth weight recorded in the MCH Handbook were analyzed, after excluding data from participants who could not refer to this Handbook. The ratio of the increase to the current weight was calculated based on birth weight. Furthermore, the percentage of the population with a low birth weight (LBW) (< 2,500 g) was calculated.

Cross-tabulations were used to calculate the percentage of responses to each of the following options: (a) *annual income*, (b) *body image, weight fluctuation, weight gain*, (c) *exercise habits*, (c-1) *reasons for exercise habits*, (c-2) *reasons for not having exercise habits*, and (d) *eating habits*. For participants who answered “yes” to (c-1) the *reasons for exercise habits*, the mean and SD were calculated for the number of days and hours of exercise per week.

#### 2.4.2. Primary survey

The primary survey was analyzed by comparing Analysis 1: underweight and normal-weight groups, and then Analysis 2: underweight non-diet experienced group (NDG) and diet experienced group (DG). For Analysis 2, respondents were first asked whether they had ever dieted, then categorized into NDG and DG, and a comparative analysis of each questionnaire item was conducted. The specific analysis design is as follows:

##### 2.4.2.1. Comparative analysis of participant background, exercise habits, and eating habits

First, for (a) *demographic data*, the mean and standard deviation (two decimal place) for each item were calculated. An unpaired t-test was conducted to compare the differences between the two groups.

Next, a χ^2^ test was conducted on the following questionnaires, and residuals were tested if significant differences were found: (a) *annual income*, (b) *Body image, weight fluctuation, weight gain*, (c) *exercise habits*, (c-1) *reasons for exercise habits*, (c-2) *reasons for not exercising*, and (d) *eating habits*. In all cases, the percentage (one decimal place) of the number of respondents for each item choice was calculated. If the answer to (c-1) *reasons for exercise habits* was “yes,” the mean and SD of the number of days and hours of exercise per week were calculated and compared using an unpaired t-test.

##### 2.4.2.2. Comparative analysis of questionnaires

For the five questionnaires (Table 1), the mean and standard deviation of the scores were calculated according to the method used to calculate the scores for each scale (two decimal places). Next, an unpaired *t*-test was conducted to compare differences between the two groups. For the EAT scale, which has an index of total scale scores, the percentage of respondents in the normal, mid-range disordered eating attitudes, and disordered eating attitudes categories was calculated (one decimal place). For the other scales, percentiles were calculated and classified as low [>25th percentile, medium group for 25-<75th, and high (≥75th percentile)]. The percentage of each percentile population was then calculated (one decimal place).

## 3. Results

### 3.1. Results of the screening survey

The screening survey’s results are presented in Table 2. The percentages of underweight, normal weight, and obesity were 23.54, 68.91, and 7.55%, respectively. The results for each question were as follows:

**Table 2:** Demographic data from screening questionnaire.

	Underweight *n* = 1,390 (23.54%) (BMI < 18.5Kg/m^2^)		Normal range *n* = 4,069 (68.91%) (BMI 18.5- < 25 Kg/m^2^)		Obese n = 446 (7.55%) (BMI ≧25 Kg/m^2^)	
	Mean	SD	Mean	SD	Mean	SD
(a) Demographic data						
Age (years)	25.07	3.13	25.13	3.14	25.70	2.89
Age at first menstruation (years)	12.57	1.60	12.27	1.48	11.62	1.37
High (cm)	158.09	5.63	157.67	5.39	157.27	5.59
Weight (kg)	43.49	3.79	51.59	5.14	69.58	10.28
BMI (Kg/m^2^)	17.38	0.90	20.74	1.59	28.09	3.57
Birth weight (g)‡ (n = 418/1,266/121)	2989.50	373.67	3043.29	381.20	3085.44	468.74
Maximum weight (kg) [BMI]	49.09 [19.62]	5.59	56.49 [22.71]	6.63	74.83 [30.20]	12.01
Ideal weight (kg) [BMI]	43.70 [17.47]	5.18	47.58 [19.12]	4.77	53.63 [21.66]	6.60
Weight perceived as unacceptable (kg) [BMI]	49.27 [19.70]	5.47	55.29 [22.23]	6.34	71.63 [28.93]	13.77
Weight perceived as model shape (kg) [BMI]	42.29 [16.91]	3.92	44.43 [17.86]	3.99	46.51 [18.81]	4.93
Weight perceived as obese (kg) [BMI]	53.03 [21.22]	6.91	57.24 [23.02]	6.65	65.02 [26.27]	8.75
Annual income (%)* ≧2,000,000 yen	35.68%		36.08%		47.31%	
2,000,000 yen~>4,000,000 yen	33.31%		33.64%		30.27%	
4,000,000 yen~>6,000,000 yen	8.99%		9.17%		4.26%	
6,000,000 yen~>8,000,000 yen	1.80%		0.88%		1.35%	
8,000,000 yen~10,000,000 yen	0.65%		0.37%		0.00%	
≧10,000,000 yen	0.43%		0.17%		0.22%	
I do not know	7.19%		8.23%		8.30%	
I prefer not to answer	11.94%		11.45%		8.30%	
(b) Body image, weight fluctuation, gaining weight†						
b2. Body image (Skinny/Normal /Obese) (%)	64.5%/27.6%/7.9%		11.5%/40.9%/47.6%		1.8%/4.00%/94.2%	
b3. Body shape satisfaction (Dissatisfied/Normal/Satisfied) (%)	21.4%/68.7%/9.9%		44.6%/52.3%/3.0%		85.0%/14.6%/0.4%	
b4. Weight fluctuation (Easy to lose/Unchanged/Easy to gain) (%)	21.2%/54.9%/23.9%		5.2%/35.5%/59.4%		1.8%/14.3%/83.9%	
b5. Want to gain more weight (Disagree/Neutral/Agree) (%)	52.1%/34.0%/13.9%		73.0%/20.4%/6.6%		80.7%/15.9%/3.4%	
(c) Exercise habits†						
c1. Exercise habits in the past year (at least once a week) (yes %)	48.3%		52.4%		50.4%	
c2. Elementary school age (yes %)	51.7%		52.4%		47.6%	
c3. Junior high school age (club activities) (yes %)	47.0%		52.7%		46.4%	
c4. High school age (club activities) (yes/no/N/A¶) (%)	26.5%/60.4%/13.1%		31.8%/55.2%/12.9%		26.5%/57.2%/16.4%	
c5. Sports to continue throughout your life (Individual/Group/None)	28.9%/7.8%/63.3%		27.9%/12.5%/59.5%		19.3%/11.7%/69.1%	
c6. Current exercise habits are important (Disagree/Neutral/Agree)	10.1%/46.8%/43.1%		9.0%/44.1%/46.9%		6.5%/37.7%/55.8%	
c7. Future exercise habits are important (Disagree/Neutral/Agree)	8.5%/42.4%/50.0%		7.6%/39.4%/53.0%		5.4%/33.6%/61.0%	
c8. Going to have an exercise routine in future (Disagree/Neutral/Agree)	11.4%/54.2%/34.4%		9.8%/49.9%/40.4%		7.2%/52.0%/40.8%	
c9. Going to take specific actions for exercise habits (Disagree/Neutral/Agree)	17.3%/59.3%/23.5%		16.5%/57.4%/26.1%		15.7%/60.3%/24.0%	
(c-1) Reason of exercise habits§ (n = 671/2,131/209) †						
c-1-1. Health and fitness (yes %)	82.1%		81.3%		75.1%	
c-1-2. Fun or Distractions (yes %)	64.8%		64.0%		51.7%	
c-1-3. To feel inadequate in physical exercise (yes %)	79.6%		80.9%		81.8%	
c-1-4. For spiritual cultivation or training (yes %)	25.5%		24.1%		25.5%	
c-1-5. To improve my record or ability (yes %)	24.4%		23.7%		15.3%	
c-1-6. To contact family (yes %)	17.9%		19.4%		17.2%	
c-1-7. Socializing with friends and colleagues (yes %)	25.5%		24.8%		24.6%	
c-1-8. Beauty and obesity reduction (yes %)	58.1%		71.8%		74.6%	
c-1-9. Club activities (yes %)	13.3%		13.9%		13.4%	
c-1-10. To relieve stress (yes %)	49.3%		53.2%		43.5%	
(c-2) Reason of not having exercise habits‖ (n = 719/1938/237) †						
c-2-1. Too busy (yes %)	60.1%		65.3%		64.3%	
c-2-2. Physically weak (yes %)	11.5%		8.2%		8.4%	
c-2-3. Old age (yes %)	15.4%		15.3%		15.9%	
c-2-4. No place or facilities (yes %)	37.7%		37.6%		47.7%	
c-2-5. Do not have friends (yes %)	39.4%		42.8%		48.5%	
c-2-6. Do not have a mentor (yes %)	18.4%		21.0%		27.8%	
c-2-7. Costs money (yes %)	44.4%		50.1%		52.7%	
c-2-8. Do not like exercise/sports (yes %)	53.7%		49.3%		63.7%	
c-2-9. Never had the chance (yes %)	60.1%		60.9%		60.8%	
(d) Eating habits†						
d3. Current eating habits are important (Disagree/Neutral/Agree) (%)	7.8%/45.3%/46.9%		8.0%/41.8%/50.3%		4.9%/38.6%/56.5%	
d4. Future eating habits are important (Disagree/Neutral/Agree) (%)	7.6%/40.4%/51.9%		7.0%/36.9%/56.1%		4.7%/35.4%/59.9%	
d5. Want to have the right eating habits (Disagree/Neutral/Agree) (%)	9.0%/48.7%/42.3%		8.2%/45.8%/46.0%		6.7%/46.9%/46.4%	
d6. Will take action to develop good eating habits (Disagree/Neutral/Agree) (%)	13.5%/57.2%/29.4%		12.7%/57.4%/30.0%		13.2%/56.3%/30.5%	
d7. Want to increase food intake (Disagree/Neutral/Agree) (%)	42.3%/45.3%/12.4%		57.5%/35.8%/6.7%		67.0%/28.5%/4.5%	

*Annual income: Salaried full-time employees who had an average salary of 4,430,000 yen (men: 5,450,000 yen; women: 3,020000 yen) (18).

† Cross tabulation results.

‡ Data for 1,805 participants recorded in the Maternal and Child Health Handbook.

¶ Not applicable (participants who have not been enrolled in high school).

#### 3.1.1. Demographic data

The mean current BMI was 17.4 for underweight women, and 20.7 and 28.1 for women with normal weight and obesity, respectively. Those who reported their birth weight recorded in their MCH handbook accounted for 30.57% (n = 1,805) of all participants, with the lowest mean for underweight being 2989.50 ± 373.67 g. The percentages of LBW were 10.6% for underweight, 8.3% for normal weight, and 9.6% for obesity. The ideal underweight group (BMI mean = 17.5) was generally consistent with their current weight. Weight perceived as unacceptable (BMI mean = 19.7) was generally consistent with the maximum weight (BMI mean = 19.6). All 3 groups generally had a mean weight (BMI), and the weight perceived as ideal was generally indicative of underweight. The underweight group’s perceived obesity (BMI mean = 21.2) corresponded to the BMI index of normal-weight group. Most annual incomes were distributed in the ≥2,000,000 yen and 2,000,000 yen – >4,000,000 yen categories.

#### 3.1.2. Body image, weight fluctuation, gaining weight

Regarding **Qb2,** about how the individual envisions their body image: 64.46% of underweight respondents answered “Skinny.” Meanwhile, 47.63% of normal-weight and 94.2% of obese respondents answered “Obese.” Regarding **Qb3**, a question about satisfaction with an individual’s body shape, 78.6% of the underweight respondents answered “Normal/Satisfied,” while 44.6% of normal-weight and 85.0% of obese respondents answered “Dissatisfied.” Regarding **Qb4,** weight fluctuation, 21.2% of the underweight respondents reported that their weight was “Easy to lose,” 59.4% of the normal-weight respondents and 83.9% of the obese respondents reported that their weight was “Easy to gain.” For **Qb5,** wanting to gain more weight, 52.1% of underweight, 73.0% of normal weight, and 80.7% of obese respondents answered “Disagree.”

#### 3.1.3. Exercise habits

Respondents for **Qc1,** a question about exercise habits in the past year, were approximately 50% for underweight, normal-weight, and obese individuals. The average number of days/weeks of exercise for underweight, normal-weight, and obese participants was 1.8 ± 1.3, 1.8 ± 1.4, and 1.7 ± 1.2 days, respectively. The amounts of hours per exercise session was 1.4 ± 1.1, 1.3 ± 1.0, and 1.1 ± 0.89 h for underweight, normal-weight, and obese respondents, respectively. Regarding past exercise habits, approximately 50% individuals in the elementary and junior high school age group each had exercise habits. In the high school age group, 26.5% were either underweight or obese, while 31.8% were of normal weight.

The participants who answered that they currently exercised (n = 3,011) commonly selected **Qc1-1,** health and fitness and **Qc1-3,** feeling inadequate in physical exercise as their **(Qc-1)** reasons for exercise habits and exhibited the highest percentages in the 70–80% range. Responses to **Qc1-8,** beauty and obesity reduction, was 58.1% for underweight, 71.8% for normal-weight, and 74.6% for obese respondents. Currently, among the respondents who reported not having exercise habits (n=2,894), the responses to Qc-2, reason for not having exercise habits, were consistent across those who were underweight, of normal weight, and obese. Both Qc2-1, too busy, and Qc2-9, never had the chance, were indicated by 60-65% of respondents. Of the respondents who selected **Qc2-8,** do not like exercise/sports, 63.7% were obese, 53.7% were underweight, and 49.3% were normal-weight.

Regarding question **Qc5,** sports to continue throughout your life, 63.3% of underweight, 59.5% of normal-weight, and 69.1% of obese respondents answered “None.” As a response to **Qc6,** current exercise habits are important and **Qc7,** future exercise habits are important, 43.1 and 50.0% of underweight respondents, 46.9 and 53.0% of normal-weight respondents, and 55.8 and 61.0% of obese respondents answered, “Agree.” For **Qc8,** going to have an exercise routine in the future, 34.4% of the respondents who were underweight, 40.4% of the respondents who had normal weight, and 40.8% of the respondents who were obese answered “Agree.” By contrast, 23.5 to 26.1% responded “Agree” to **Qc-9,** going to take specific actions for exercise habits.

#### 3.1.4. Eating habits

The following percentages of respondents answered “Agree” on **Qd3,** current eating habits are important, and **Qd4,** future eating habits are important: 46.9 and 51.9% for underweight, 50.3 and 56.1% for normal-weight, and 56.5 and 59.9% for obese individuals, respectively. Percentages of respondents who answered “Agree” on **Qd5,** want to have the right eating habits, 42.3% of the respondents were underweight, 46.0% were a normal weight, and 46.4% were obese. By contrast, the percentage of respondents who answered “Agree” to **Qd6,** will take action to develop good eating habits, 29.4% were underweight, 30.0% were normal-weight, and 30.5% were obese participants. Moreover, the percentage of respondents who answered “Agree” to **Qd7,** want to increase food intake, 42.3% were underweight, 57.5% were normal-weight, and 67.0% were obese women.

### 3.2. Analysis 1: comparative results in the underweight and normal-weight categories

#### Comparing participant background, exercise habits, and eating habits

Table 3 presents the comparison results of the participants’ backgrounds, exercise habits, and eating habits between underweight and normal-weight participants. Each questionnaire’s results are as follows.

**Table 3:** Comparison of questionnaire among underweight and normal-weight Women (*t*-test/chi-square test).

	Underweight *n* = 400		Normal range *n* = 189		*t*	*p*
	Mean	SD	Mean	SD		
(a) Demographic Data						
Age (years)	25.03	3.04	25.49	3.04	−1.723	0.085
Age at first menstruation (years)	12.67	1.58	12.39	1.43	2.038	0.042
Height (cm)	158.73	5.96	158.27	5.15	0.961	0.337
Weight (kg)	43.96	3.99	51.78	5.10	−18.572	<0.001
BMI (Kg/m^2^)	17.43	0.92	20.65	1.55	−26.474	<0.001
Birth weight (g)‡ (n = 304/189)	2982.58	372.77	3030.47	413.78	−1.329	0.092
Maximum weight (kg)[BMI]	49.64 [19.69]	5.4756	56.70 [22.60]	7.2078	−11.931	<0.001
Ideal weight (kg)[BMI]	43.99 [17.45]	4.1112	48.10 [19.19]	4.176	−11.282	<0.001
Weight perceived as unacceptable (kg) [BMI]	49.60 [19.67]	5.3845	56.73 [22.62]	7.7233	−11.449	<0.001
Weight perceived as model shape (kg) [BMI]	42.60 [16.89]	4.1598	44.79 [17.88]	3.816	−6.120	<0.001
Weight perceived as obese (kg) [BMI]	52.95 [21.01]	6.7498	58.40 [23.32]	7.0286	−9.034	<0.001
Age at which weight gain became a concern (years)§ (*n* = 321/171)	19.22	4.85	18.94	5.58	0.560	0.576
Age decided to diet (years) ‖ (n = 220/135)	17.45	3.70	17.99	4.09	−1.277	0.203
Annual income * (yes) (%) †					*χ* ^**2**^	*p*
≧2,000,000 yen	34.5%		36.0%		3.940	0.685
2,000,000 yen~>4,000,000 yen	31.8%		32.3%			
4,000,000 yen~>6,000,000 yen	10.0%		13.2%			
6,000,000 yen~>8,000,000 yen	3.5%		3.2%			
8,000,000 yen~>10,000,000 yen	1.8%		0.5%			
I do not know	7.5%		5.3%			
I prefer not to answer	11.0%		9.5%			
(b) Diet experience, Body image, weight fluctuation, gaining weight^†^					*χ* ^2^	*p*
b1. Diet experience (yes) (%)	55.00%		71.43%		14.467	<0.001
b2. Body image (Skinny/Normal /Obese) (%)	62.5%**/29.0%/8.5%		12.2%/42.3%**/45.5%**		163.261	<0.001
b3. Body shape satisfaction (Dissatisfied/Normal/Satisfied) (%)	19.5%/68.0%**/12.5%**		42.9%**/52.9%/4.2%		39.476	<0.001
b4. Weight fluctuation (Easy to lose/Unchanged/Easy to gain) (%)	18.5%**/52.8%**/28.7%		5.3%/35.4%/59.3%**		54.842	<0.001
b5. Want to gain more weight (Disagree/Neutral/Agree) (%)	54.5% /30.5%/15.0%*		66.7%**/25.4%/7.9%		9.440	<0.001
(c) Exercise habits†					*χ* ^2^	*p*
c1. Exercise habits in the past year (at least once a week) (yes %)	51.7%		50.30%		0.113	0.736
c2. Elementary school age (yes %)	53.0%		52.40%		0.020	0.888
c3. Junior high school age (club activities) (yes %)	48.50%		55.60%		2.556	0.110
c4. High school age (club activities) (yes/no/N/A¶) (%)	30.0%/57.8%/12.3%		27.0%/58.2%/14.8%		1.052	0.591
c5. Sports to continue throughout your life (Individual/Group/None)	34.5%/7.5%/58.0%		28.6%/12.7%/58.7%		5.179	0.075
c6. Current exercise habits are important (Disagree/Neutral/Agree)	9.0%/43.0%/48.0%		13.8%/46.6%/39.7%		5.086	0.079
c7. Future exercise habits are important (Disagree/Neutral/Agree)	7.2%/41.5%/51.2%		9.0%/41.8%/49.2%		0.61	0.737
c8. Going to have an exercise routine in future (Disagree/Neutral/Agree)	12.0%/51.2%/36.8%		12.7%/51.3%/36.0%		0.07	0.965
c9. Going to take specific actions for exercise habits (Disagree/Neutral/Agree)	15.8%/56.3%/28.0%		17.5%/58.7%/23.8%		0.07	0.965
(c-1) Reasons for exercise habits§ (n = 210/92) †					*χ^2^*	*p*
c-1-1. Health and fitness (yes %)	87.1%		82.1%		1.517	0.218
c-1-2. Fun or Distractions (yes %)	72.0%		66.3%		0.999	0.318
c-1-3. To feel inadequate in physical exercise (yes %)	81.6%		82.1%		0.009	0.923
c-1-4. For spiritual cultivation or training (yes %)	31.4%		28.4%		0.273	0.601
c-1-5. To improve my record or ability (yes %)	27.5%		30.5%		0.286	0.593
c-1-6. To contact family (yes %)	16.9%		24.2%		2.238	0.135
c-1-7. Socializing with friends and colleagues (yes %)	25.1%		26.3%		0.049	0.825
c-1-8. Beauty and obesity reduction (yes %)	61.4%		68.4%		1.41	0.236
c-1-9. Club activities (yes %)	9.7%		12.6%		0.61	0.436
c-1-10. To relieve stress (yes %)	52.7%		48.4%		0.47	0.494
(c-2) Reasons for not having exercise habits (*n* = 190/97)^†^					*χ* ^2^	*p*
c-2-1. Too busy (yes %)	58.5%		61.7%		0.261	0.609
c-2-2. Physically weak (yes %)	10.9%		9.6%		0.115	0.734
c-2-3. Old age (yes %)	15.0%		13.8%		0.072	0.788
c-2-4. No place or facilities (yes %)	39.4%		29.8%		2.517	0.113
c-2-5. Do not have friends (yes %)	36.3%		31.9%		0.528	0.467
c-2-6. Do not have a mentor (yes %)	14.5%		17.0%		0.308	0.579
c-2-7. Costs money (yes %)	43.5%		42.6%		0.024	0.876
c-2-8. Do not like exercise/sports (yes %)	57.5%		54.3%		0.273	0.601
c-2-9. Never had the chance (yes %)	60.6%		60.6%		0.000	0.998
(d) Eating habits†					*χ* ^2^	*p*
d1. Current food intake is good (Good/Neutral/Problematic) (%)	40.5%/31.5%/28.0%		43.4%/28.0%/28.6%		0.778	0.678
d2. I get the nutrients needed from my daily diet (Adequate/Sufficient/Insufficient) (%)	5.8%/60.8%/33.5%		5.8%/59.8%/34.4%		0.051	0.975
d3. Current eating habits are important (Disagree/Neutral/Agree) (%)	6.8%/42.8%/50.5%*		13.2%** /45.5%/41.3%		8.623	0.013
d4. Future eating habits are important (Disagree/Neutral/Agree) (%)	5.8%/38.5%/55.8%		11.1%*/40.2%/48.7%		6.235	0.044
d5. Want to have the right eating habits (Disagree/Neutral/Agree) (%)	9.0%/43.0%/48.0%*		12.7%/49.7%/37.6%		6.142	0.046
d6. Will take action to develop good eating habits (Disagree/Neutral/Agree) (%)	10.5%/55.8%/33.8%**		19.0%**/60.3%/20.6%		15.023	<0.001
d7. Want to increase food intake (Disagree/Neutral/Agree) (%)	41.5%/45.5%/13.0%*		51.9%*/41.8%/6.3%		8.690	0.013
d8. Frequency of missing meals (Every day/Several times a week/None at all) (%)	15.9%/25.4%/58.7%		17.0%/19.1%/63.8%		0.533	0.766
d10. Stress and fatigue changed appetite (Decrease/No change/Over-eat) (%)	28.5%/33.5%/38.0%		21.7%/33.9%/44.4%		3.595	0.166

*Annual income: Salaried full-time employees who had an average salary of 4,430,000 yen (men: 5,450,000 yen; women: 3,020000 yen) (18).

† Chi-square test results. *p < 0.05, **p < 0.01.

‡ Data for 493 participants recorded in the Maternal and Child Health Handbook.

§ Data from 492 respondents who were concerned about weight gain.

‖ Data from 189 respondents who have diet experience.

¶ N/A is not applicable (participants who have not been enrolled in high school).

#### 3.2.1. Demographic data

The results are presented for the main items for which there were significant differences. The age at first menarche was slightly younger in the normal-weight group (*p* < 0.042), and the mean current BMI was lower in the underweight group (*p* < 0.001). The highest historical weight, ideal weight, unacceptable weight, and weight considered obese were 5–7 kg lower in the underweight group (*p* < 0.001), respectively. The weight considered as a model body shape was 2kg less for those who were underweight (*p* < 0.001), and the BMI was 16.9kg/m^2^, which is below the standard for underweight. Overall, 492 participants indicated that they were concerned that they would begin gaining weight.

There was no significant difference in the age at which they reported having begun to be concerned about weight gain. All were approximately 19 years old. Of the 355 underweight and normal-weight participants who indicated that they had experienced a diet, there was no significant difference in the age at which they decided to lose weight. All were approximately 17 years old. Most annual incomes were distributed in the ≥2,000,000 yen and 2,000,000 yen – >4,000,000 yen categories. No significant differences were found between the two groups.

#### 3.2.2. Diet experience, body image, weight fluctuation, gaining weight

Regarding the **Qb1,** diet experience, the normal-weight group exhibited a significantly higher response rate (71.4%, *p* < 0.001). For **Qb2,** body image, underweight respondents were more likely to answer “Skinny” (62.5%, *p* < 0.01), and normal-weight respondents were more likely to answer “Obese” (45.5%, *p* < 0.01). Regarding question **Qb3,** body shape satisfaction, “Normal” was selected significantly more frequently by the underweight group (68.0%, *p* < 0.01), and “Dissatisfied” was selected significantly more often by normal-weight participants (42.9%, *p* < 0.01). For the **Qb4,** weight fluctuation question, “Easy to lose” and “Unchanged” were significantly more frequently provided by the underweight group (18.5 and 52.8%, respectively; *p* < 0.01). For Respondents who answered **Qb5**, want to gain more weight, “Agree” was significantly more frequent in the underweight group (15.0%, *p* < 0.01), and “Disagree” was significantly higher in the normal-weight group (66.7%, *p* < 0.01).

#### 3.2.3. Exercise habits

The percentage of respondents who reported exercise habits in the past year and exercise habits in elementary school and junior high school, were approximately 48–55% for both underweight and normal-weight groups, and 20–30% for high school aged participants, with no significant differences. Among the 302 respondents who answered that they had **Qc1**, exercise habits in the past year, the average number of exercise days per week for those who were underweight (*n* = 207) was 2.8 ± 2.0 days, and for those of normal weight (*n* = 95) it was 2.4 ± 1.5 days, with no significant difference between the two groups. For the duration of exercise per session, the underweight group (1.1 ± 1.2 h) was significantly greater than the normal-weight group (0.8 ± 0.8 h; *p* < 0.05). The proportion of responses to **(Qc-1),** reasons for exercise habits, was not significantly different between the groups for all items. The 287 participants who answered no to **Qc1,** exercise habits in the past year, there was no significant difference between the groups for all items of **(Qc-2),** reasons for not having exercise habits. There were no significant differences between the underweight and normal-weight groups regarding the questions perceptions and behaviors related to exercise habits (**Qc3–6**). In all cases, “Disagree” responses ranged from 9 to 17.5%, with most responding “Neutral/Agree.”

#### 3.2.4. Eating habits

The response rate to **Qd1,** current food intake is good, was approximately 71% for “Good/Neutral” for both underweight and normal-weight respondents. Similarly, 65–66% of the respondents answered “Adequate/Roughly enough” for **Qd2,** I get the nutrients needed from my daily diet; there was no significant difference. By contrast, for **Qd3,** current eating habits are important, the percentage of responses to “Agree” was higher for the underweight group (50.5%, *p* < 0.05). Most individuals from both groups responded “Neutral/Agree” to these questions. For the **Qd4,** future eating habits are important, 11.1% of the normal-weight group answered “Agree,” approximately twice as much as the underweight group (*p* < 0.05). Most individuals from both groups responded “Neutral/Agree. Regarding **Qd5,** want to have the right eating habits, the “Agree” response was significantly higher in the underweight group (48.0%, *p* < 0.05). For **Qd6,** will take action to develop good eating habits, underweight respondents were more likely to respond “Agree” (33.8%, *p* < 0.01), and normal-weight respondents were more likely to respond “Disagree” (19.0%, *p* < 0.01). For **Qd7,** want to increase food intake, “Agree” was selected more frequently by underweight respondents (13.0%, *p* < 0.05), while “Disagree” was selected more frequently by normal-weight respondents (51.9%, *p* < 0.05). For **Qd8,** frequency of missing meals, 15.9% of the underweight group and 17.0% normal-weight group responded, with no significant difference between the groups. There was also no difference in **Qd9,** stress and fatigue change appetite. Further, 28.5% for the underweight group and 21.7% for the normal-weight group answered “Decrease,” and 38.0% for underweight and 44.4% for normal-weight answered “Overeat.”

#### 3.2.5. Comparison of questionnaires

Table 4 presents the results of a comparison of the five questionnaires. There were no significant differences between the underweight and normal-weight groups on the EAT-26, and the mean scores were all within the eight-point range. Overall, 16.3% of underweight and 11.5% of normal-weight respondents had midrange eating disorders (9–20 points), and 17.5 and 11.1% had severe eating disorders (≥ 20 points). No significant differences were found for eHealth, with underweight and normal-weight groups scoring approximately 23 points. The SATAQ-3 JS scored approximately 33 to 34 points with underweight and normal-weight groups, which was not significantly different. The lower scale of SATAQ-3 JS was significantly different, but only for the category *Pressures*, with an increased rate of normal weight (0.97 points, *p* < 0.01). Concerning TIPI-J, scores for the category *Extraversion* were 0.74 points higher for the normal-weight group (*p* < 0.01). For the category *Openness*, the normal-weight group scored 0.44 points higher (*p* < 0.01). Regarding RSES, there was no significant difference, and the mean score for both underweight and normal-weight groups was approximately 29 points.

**Table 4:** Comparison of standardized questionnaire items and subscales among underweight and normal-weight women.

Questionnaires and subscales	Underweight *n* = 400			Normal range *n* = 189			Median (IQR)	*t*	*df*	*p*	95% CI
	Mean	SD	Low/Middle/High (*n*%)	Mean	SD	Low/Middle/High (*n*%)					
Eating Attitude Test (EAT-26)	8.62	11.76	72.3%/16.32%/11.5%	8.47	10.99	68.3%/20.6%/11.1%	5 (1–11)	0.149	587.00	0.881	−1.845 ~ 2.149
eHealth Literacy Scale (eHEALTH)	22.91	7.07	24.0%/53.0%/23.0%	23.71	7.76	20.6%/52.4%/27.0%	24 (18–28)	−1.257	587.00	0.209	−2.074 ~ 0.455
Sociocultural Attitudes Towards Appearance Questionnaire-3 (SATAQ-3 JS)	33.05	13.03	24.0%/55.3%/20.8%	34.44	14.14	24.0%/55.3%/20.8%	34 (23–43)	−1.179	587.00	0.239	−3.716 ~ 0.928
SATAQ Pressures	7.10	3.99	0%/81.5%/18.5%	8.07	4.36	0%/74.1%/25.9%	6 (3–11)	−2.661	587.00	0.008	−1.679 ~ −0.253
SATAQ Internalization General	9.65	4.39	19.5%/56.5%/24.0%	9.98	4.41	18.5%/56.1%/25.4%	10 (6–12)	−0.834	587.00	0.405	−1.086 ~ 0.439
SATAQ Internalization-Athlete	7.17	3.49	0%/81.5%/18.5%	7.44	3.93	0%/76.7%/23.3%	7 (3–10)	−0.835	332.35	0.404	−0.938 ~ 0.379
SATAQ Information	9.12	4.13	23.3%/57.0%/19.8%	8.95	4.24	24.3%/58.7%/16.9%	9 (6–12)	0.477	587.00	0.633	−0.546 ~ 0.897
Ten-Item Personality Inventory (TIPI-J)											
TIPI-J Extraversion	6.87	2.76	20.8%/62.5%/16.8%	7.61	2.73	20.8%/62.5%/16.8%	7 (5–9)	−3.039	587.00	0.002	−1.216 ~ −0.261
TIPI-J Agreeableness	9.43	2.29	19.5%/60.0%/20.5%	9.15	2.22	19.6%/65.1%/15.3%	9 (8–11)	1.382	587.00	0.167	−0.117 ~ 0.670
TIPI-J Conscientiousness	7.75	2.48	19.0%/58.8%/22.3%	7.48	2.66	21.2%/58.7%/20.1%	8 (6–9)	1.198	587.00	0.231	−0.172 ~ 0.709
TIPI-J Emotional Stability	8.88	2.37	24.3%/52.5%/23.3%	8.84	2.52	23.8%/53.4%/22.8%	9 (8–10)	0.195	587.00	0.846	−0.377 ~ 0.460
TIPI-J Openness	7.12	2.41	26.0%/61.0%/13.0%	7.56	2.47	19.6%/63.0%/17.5%	8 (6–9)	−2.042	587.00	0.042	−0.860 ~ 0–0.017
Rosenberg’s Self Esteem Scale (RSES)†	28.64	7.85	26.0%/50.2%/23.8%	29.20	7.63	21.7%/52.9%/25.4%	29 (25–33)	−0.817	587.00	0.414	−1.909 ~ 0.788

IQR: Interquartile Range.

† The Japanese version is a 5-point Likert scale including intermediate item 3.

Low/Middle/High (%): Low refers to the percentage of the population below the 25th percentile, middle refers to the IQR, and High refers to the 75th percentile and above.

### 3.3. Analysis 2: comparative results in the underweight dieting experience

#### Comparison of participant background, exercise habits, and eating habits

Table 5 presents the comparison results of the participants’ backgrounds, exercise habits, and eating habits between NDG and DG respondents. The results of each questionnaire are as follows:

**Table 5:** Comparison of questionnaire among underweight women’s dieting experience.

	Underweight women diet experience				*t*	*p*
	Non-experience *n* = 180		Experienced *n* = 220			
	Mean	SD	Mean	SD		
(a) Demographic data						
Age (years)	24.97	3.03	25.08	3.05	−0.377	0.706
Age at first menstruation (years)	12.70	1.63	12.64	1.54	0.37	0.710
Height (cm)	158.64	6.09	158.81	5.87	−0.276	0.783
Weight (kg)	43.81	3.95	44.09	4.02	−0.700	0.484
BMI (Kg/m^2^)	17.38	0.83	17.46	0.99	−0.864	0.388
Birth weight (g)‡ (*n* = 136/168)	2894.66	396.66	3053.74	336.96	−3.780	<0.001
Maximum weight (kg) [BMI]	48.25 [19.15]	5.36	50.78 [20.12]	5.31	−4.719	<0.001
Ideal weight (kg) [BMI]	44.64 [17.72]	4.26	43.46 [17.22]	3.92	2.895	0.004
Weight perceived as unacceptable (kg) [BMI]	50.36 [19.99]	5.98	48.97 [19.41]	4.77	2.532	0.012
Weight perceived as model shape (kg) [BMI]	42.90 [17.03]	4.66	42.35 [16.78]	3.69	1.306	0.193
Weight perceived as obese (kg) [BMI]	54.31 [21.57]	7.59	51.83 [20.55]	5.76	3.622	<0.001
Age at which weight gain became a concern (years) § (*n* = 108/213)	19.95	4.93	18.85	4.77	1.93	0.055
Age decided to diet (years) ‖	—	—	17.45	3.70	—	—
Annual income* (%)†					*χ* ^2^	*p*
≧2,000,000 yen	33.9%		35.0%		7.996	0.238
2,000,000 yen~>4,000,000 yen	32.8%		30.9%			
4,000,000 yen~>6,000,000 yen	7.2%		12.3%			
6,000,000 yen~>8,000,000 yen	3.9%		3.2%			
8,000,000 yen~>10,000,000 yen	0.6%		2.7%			
I do not know	7.8%		7.3%			
I prefer not to answer	13.9%		8.6%			
(b) Body image, weight fluctuation, gaining weight†					*χ* ^2^	*p*
b2. Body image (Skinny/Normal /Obese) (%)	73.9%**/22.2%/3.9%		53.2%/34.5%**/12.3%**		20.163	<0.001
b3. Body shape satisfaction (Dissatisfied/Normal/Satisfied) (%)	15.0%/71.1%/13.9%		23.2%/65.5%/11.4%		4.369	0.113
b4. Weight fluctuation (Easy to lose/Unchanged/Easy to gain) (%)	26.1%**/63.9%**/10.0%		12.3%/43.6%/44.1%**		57.966	<0.001
b5. Want to gain more weight (Disagree/Neutral/Agree) (%)	38.9%/41.1%**/20.0%*		67.3%**/21.8%/10.9%		32.171	<0.001
(c) Exercise habits†					*χ* ^2^	*p*
c1. Exercise habits in the past year (at least once a week) (yes %)	41.7%		60.0%		13.326	<0.001
c2. Elementary school age (yes %)	41.1%		62.7%		18.570	<0.001
c3. Junior high school age (club activities) (yes %)	40.0%		55.5%		9.467	0.002
c4. High school age (club activities) (yes/no/N/A¶) (%)	22.2%/61.1%/13.3%		33.6%/55.0%/11.4%		3.109	0.211
c5. Sports to continue throughout your life (Individual/Group/None)	25.0%/4.4%/70.6%**		42.3%**/10.0%*/47.7%		21.530	<0.001
c6. Current exercise habits are important (Disagree/Neutral/Agree)	10.0%/50.0%*/40.0%		8.2%/37.3%/54.5%**		8.457	0.015
c7. Future exercise habits are important (Disagree/Neutral/Agree)	10.0%/45.6%*/44.4%		6.8%/33.6%/59.5%**		9.10	0.011
c8. Going to have an exercise routine in future (Disagree/Neutral/Agree)	12.8%/58.9%*/28.3%		9.5%/46.4%/44.1%**		10.57	0.005
c9. Going to take specific actions for exercise habits (Disagree/Neutral/Agree)	18.9%*/61.7%/19.4%		10.5%/52.7%/36.8%**		16.64	<0.001
(c-1) Reasons for exercise habits§ (n = 75/132) †					*χ* ^2^	*p*
c-1-1. Health and fitness (yes %)	84.0%		89.4%		1.267	0.260
c-1-2. Fun or Distractions (yes %)	72.0%		72.0%		0.000	0.996
c-1-3. To feel inadequate in physical exercise (yes %)	73.3%		86.4%		5.418	0.020
c-1-4. For spiritual cultivation or training (yes %)	20.0%		37.9%		7.097	0.008
c-1-5. To improve my record or ability (yes %)	25.3%		28.8%		0.286	0.593
c-1-6. To contact family (yes %)	17.3%		16.7%		0.015	0.902
c-1-7. Socializing with friends and colleagues (yes %)	32.0%		21.2%		2.959	0.085
c-1-8. Beauty and obesity reduction (yes %)	44.0%		71.2%		14.94	<0.001
c-1-9. Club activities (yes %)	12.0%		8.3%		0.74	0.391
c-1-10. To relieve stress (yes %)	54.7%		51.5%		0.19	0.662
(c-2) Reasons for not having exercise habits (*n* = 105/88) †					*χ* ^2^	*p*
c-2-1. Too busy (yes %)	52.4%		65.9%		3.610	0.057
c-2-2. Physically weak (yes %)	11.4%		10.2%		0.071	0.790
c-2-3. Old age (yes %)	13.3%		17.0%		0.517	0.472
c-2-4. No place or facilities (yes %)	38.1%		40.9%		0.159	0.690
c-2-5. Do not have friends (yes %)	32.4%		40.9%		1.506	0.220
c-2-6. Do not have a mentor (yes %)	13.3%		15.9%		0.256	0.613
c-2-7. Costs money (yes %)	40.0%		47.7%		1.163	0.281
c-2-8. Do not like exercise/sports (yes %)	57.1%		58.0%		0.013	0.910
c-2-9. Never had the chance (yes %)	62.9%		58.0%		0.482	0.488
(d) Eating habits					*χ* ^2^	*p*
d1. Current food intake is good (Good/Neutral/Problematic) (%)	43.9%/27.2%/28.9%		37.7%/35.0%/27.3%		2.922	0.232
d2. I get the nutrients needed from my daily diet (Adequate/Sufficient/Insufficient) (%)	32.8%/60.6%/6.7%		34.1%/60.9%/5.0%		0.531	0.767
d3. Current eating habits are important (Disagree/Neutral/Agree) (%)	7.8%/47.8%/44.4%		5.9%/38.6%/55.5%		4.824	0.090
d4. Future eating habits are important (Disagree/Neutral/Agree) (%)	5.6%/45.6%**/48.9%		5.9%/32.7%/61.4%*		7.017	0.030
d5. Want to have the right eating habits (Disagree/Neutral/Agree) (%)	9.4%/48.3%/42.2%		8.6%/38.6%/52.7%		4.513	0.105
d6. Will take action to develop good eating habits (Disagree/Neutral/Agree) (%)	10.6%/62.2%*/27.2%		10.5%/50.5%/39.1%*		6.592	0.037
d7. Want to increase food intake (Disagree/Neutral/Agree) (%)	32.2%/52.8%**/15.0%		49.1%**/39.5%/11.4%		11.605	0.003
d8. Frequency of missing meals (Every day/Several times a week/None at all) (%)	17.2%/25.0%/57.8%		15.5%/30.5%/54.1%		1.484	0.476
d10. Stress and fatigue appetite change (Decrease/No change/Over-eat) (%)	34.4%*/41.7%**/23.9%		23.6%/26.8%/49.5%**		27.723	<0.001

*Annual income: Salaried full-time employees who had an average salary of 4,430,000 yen (men: 5,450,000 yen; women: 3,020000 yen) (18).

† Chi-square test results. *p < 0.05, **p < 0.01.

‡ Data for 493 participants recorded in the Maternal and Child Health Handbook.

§ Data from 321 respondents who were concerned about weight gain.

‖ Data from 189 respondents who have diet experience.

¶ N/A is not applicable (participants who have not been enrolled in high school).

#### 3.3.1. Demographic data

The mean current body mass index (BMI) of the NDG was 43.8 ± 4.0 kg (17.4) and 44.1 ± 4.0 kg (17.4) for the DG. For birth weight, the NDGs exhibited as low as 2894.7 ± 396.7 g (*p* < 0.001). The maximum weight (BMI) was significantly lower in the NDG group (BMI = 19.2, *p* < 0.001). For ideal weight, NDGs indicated more weight than current weight (BMI = 17.7, *p* < 0.01). Weight perceived as unacceptable and weight perceived as obese were significantly less common in DG, BMI = 19.4 and 20.6 (*p* < 0.001). Further, 321 participants indicated that they had always been concerned about gaining weight. There was no significant difference in the age at which they began to be concerned about gaining weight, with NDGs in their 20s and DGs in their 18 s; the average age at which DGs decided to lose weight was 17 years old. Most annual incomes were distributed in the ≥2,000,000 yen and 2,000,000 yen – >4,000,000 yen categories. No significant differences were found between the two groups.

#### 3.3.2. Body image, weight fluctuation, gaining weight

For **Qb2,** body image, “Skinny” was more frequent in the NDG (73.9%, *p* < 0.01). The “Normal/Obese” response was higher in the DG (34.5 and 12.3%, *p* < 0.01). No significant difference was found for **Qb3,** body shape satisfaction, with 85.0% of NDG and 76.8% of DG responding to “Normal/Satisfied.” For the **Qb4,** weight fluctuation, “Easy to lose/Unchanged,” was more frequent in the NDG (26.1% vs. 63.9%, *p* < 0.01). “Easy to gain” was more frequent in DG (44.1%, *p* < 0.01). For **Qb5,** want to gain more weight, “Disagree” was more frequent by the DG (67.3%; *p* < 0.01), while “Neutral” was more often by the NDG (44.1%, *p* < 0.01), and the same for “Agree” (20.0%, *p* < 0.05).

#### 3.3.3. Exercise habits

Exercise habits in the past year were significantly lower in the NDGs (41.7%, *p* < 0.001). Similarly, NDGs were 21.6% less likely to exercise in the past year, and the same was true for those in elementary school (*p* < 0.001) and junior high school (*p* < 0.01). For participants who answered “yes” to **Qc1,** exercise habits in the past year (n = 207), NDG (n = 75) experienced 2.3 ± 1.6 days per week, and DG (n = 132) experienced 3.2 ± 2.1 days. The DG had more exercise days per week (*p* 0.05). The mean duration of exercise was 1.2 ± 1.3 h for the NDG and 1.1 ± 1.2 h for the DG, with no difference between the groups. There were significant differences in **Qc-1,** reasons for exercise habits for three items; **Qc1-3,** to feel inadequate in physical exercise was felt in the NDG (73.3%, *p* < 0.05), and **Qc1-4,** for spiritual cultivation or training was similarly less frequent (20.0%, *p* < 0.001). For **Qc1-8,** beauty and obesity reduction, this was significantly more frequent in the DG (70.6%, *p* < 0.001).

No significant differences were found in **Qc-2,** reasons for not having exercise habits, among participants who answered “no” to **Qc1,** exercise habits in the past year (N = 193). Similar to both groups, **Qc2-1,** too busy (approximately 5–66%), **Qc2-8,** do not like exercise/sports (around 57–58%), and **Qc2-7,** never had the chance (around 58–62%) tended to be more frequent across all items. For **Qc5,** sports to continue throughout your life, had a significantly higher percentage of DG responses, with “Individual” at 25.0% (*p* < 0.01) and “Group” at 10.0% (*p* < 0.05). The percentage of responses to “None” was significantly higher for the NDG (70.6%, *p* < 0.01).

Next, we examined questions on perceptions and behaviors related to exercise habits. For **Qc6,** current exercise habits are important, this was answered “Agree” more often by DG (54.5%, *p* < 0.01). For **Qc7,** future exercise habits are important, and **Qc8,** going to have an exercise routine in the future, these were more frequent in the DG (59.5 and 44.5%, respectively). For **Qc9,** going to take specific actions for exercise habits, this was also significantly more frequent in the DG (36.8%, *p* < 0.01); however, it was less than 40%.

#### 3.3.4. Eating habits

Regarding **Qd1,** current food intake is good, 71–73% of the respondents common in both groups answered “Good/Neutral.” Similarly, for **Qd2,** I get the nutrients I need from my daily diet; approximately 93–95% of the respondents answered: “Adequate/Roughly enough.” Furthermore, approximately 92–94% of the respondents in both groups answered “Neutral/Agree” to **Qd3,** current eating habits are important. For **Qd4,** future eating habits are important, “Agree” responses were significantly higher in the DG (61.4%, *p* < 0.01). Approximately 95% of both groups answered “Neutral/Agree” to these questions. Regarding **Qd5,** want to have the right eating habits: there was no significant difference between the two groups, with around 90–91% of both groups answering “Neutral/Agree.” For **Qd6,** will take action to develop good eating habits, it was answered significantly more often by DG (39.1%, *p* < 0.05) with “Agree” and “Neutral.” The response to “Neutral” was significantly more frequent in the NDG (62.2%, *p* < 0.05). For **Qd7,** want to increase food intake, responses as “Neutral” were significantly higher for NDG (52.8%, *p* < 0.01). “Disagree” was significantly more frequent in DG (49.1%, *p* < 0.01). For **Qd8,** frequency of missing meals, the answers were not significantly different, with 17.2% of NDG and 15.5% of DG selecting “Every day.” Regarding **Qd9,** stress and fatigue change appetite, they were significantly frequent for NDG, with 34.4% (*p* < 0.05) and 41.7% (*p* < 0.01) of “Decrease/No change being most prevalent. “Over-eat” was significantly more frequent in DG (49.5%, *p* < 0.05).

#### 3.3.5. Comparing questionnaires

Comparing the results of the five questionnaires is presented in Table 6. First, for the EAT-26, the DG score was 4.63 points higher (*p* < 0.001), which qualifies as a midrange eating disorder (10.70 ± 12.75). The NDG score was <9 points. The percentages of midrange eating disorder (9 to <20 points) and severe eating disorder (≥ 20 points) were 11.67 and 6.66% for the NDG and 20.00 and 15.45% for the DG, respectively. For eHEALTH, the DG was significantly higher by 2.48 points (*p* < 0.001). The SATAQ-3 JS had a significantly higher total score of 6.23 points for the DG (*p* < 0.001). For the subscales, DG scored significantly higher on all items (*p* < 0.001); for general internalization, DG scored over 10 points. Concerning TIPI-J, the DG scored 0.56 points higher than the NDG for *Conscientiousness* (*p* < 0.01). For *Openness*, the NDG scored 0.61 points higher (*p* < 0.05). No significant differences were observed for the other items. No significant differences were found for RSES, with mean scores of approximately 29 points for both groups noted; the percentages of low (< 24 points) and high (≥ 33 points) RSES groups were 21.1 and 25.6% for the NDG, and 25.5 and 22.3% for the DG, respectively.

**Table 6:** Comparison of standardized questionnaire items and subscales among underweight women’s dieting experience.

Questionnaires and subscales	Underweight women diet experience						Median (IQR)	*t*	*df*	*P*	95% CI
	Non-experience *n* = 180			Experienced *n* = 220							
	Mean	SD	Low/Middle/High (*n*%)	Mean	SD	Low/Middle/High (*n*%)					
Eating Attitude Test (EAT-26)	6.07	9.88	81.7%/11.7%/6.6%	10.70	12.75	64.6%/20.06%/15.5%	5 (1–11)	−4.089	396.89	<0.001	−6.852756 ~ −2.40280
eHealth Literacy Scale (eHEALTH)	21.54	7.39	32.8%/49.4%/17.8%	24.02	6.60	16.8%/55.9%/27.3%	24 (18–28)	−3.508	362.59	<0.001	−3.876 ~ −1.091
Sociocultural Attitudes Toward Appearance Questionnaire-3 (SATAQ-3 JS)	29.62	12.72	35.0%/48.9%/16.1%	35.85	12.63	16.8%/52.3%/30.9%	34 (23.5–42)	−4.895	398.00	<0.001	−8.737 ~ −3.730
SATAQ Pressures	6.22	3.85	0%/82.2%/17.8%	7.82	3.97	0%/72.7%/27.3%	6 (3–10)	−4.067	398.00	<0.001	−3.730 ~ −0.827
SATAQ Internalization General	8.46	4.29	28.9%/56.7%/14.4%	10.63	4.24	11.8%/56.4%/31.8%	10 (6–12)	−5.065	398.00	<0.001	−3.013 ~ −1.328
SATAQ Internalization-Athlete	6.53	3.26	30.6%/53.3%/16.1%	7.69	3.59	20.5%/59.1%/20.5%	7 (3.25–10)	−3.348	398.00	<0.001	−1.839 ~ −0.478
SATAQ Information	8.41	4.17	30.6%/52.8%/16.7%	9.71	4.01	17.3%/60.5%/22.3%	9 (6–12)	−3.178	398.00	<0.001	−2.110 ~ −0.497
Ten-Item Personality Inventory (TIPI-J)											
TIPI-J Extraversion	7.02	2.63	18.9%/63.3%/17.8%	6.75	2.87	22.3%/61.8%/15.9%	9 (5–9)	0.997	398.00	0.319	−0.269 ~ 0.823
TIPI-J Agreeableness	9.24	2.23	20.6%/62.8%/16.7%	9.58	2.34	18.6%/57.7%/23.6%	9 (8–11)	−1.471	398.00	0.142	−0.791 ~ 0.114
TIPI-J Conscientiousness	7.44	2.30	22.8%/60.6%/16.7%	8.00	2.60	15.9%/57.3%/26.8%	8 (6–9)	−2.28	398.00	0.012	−1.053 ~ −0.078
TIPI-J Emotional Stability	8.92	2.30	25.6%/50.6%/23.9%	8.84	2.42	23.2%/54.1%/22.7%	9 (8–10)	0.34	398.00	0.733	−0.387 ~ 0.549
TIPI-J Openness	7.45	2.41	11.7%/36.7%/51.7%	6.85	2.39	17.3%/37.7%/45.0%	8 (5–7)	2.47	398.00	0.014	0.122 ~ 1.069
Rosenberg’s Self Esteem Scale (RSES)†	29.22	7.64	21.1%/53.3%/25.6%	28.16	8.00	25.5%/52.3%/22.3%	29 (24–33)	1.34	398.00	0.180	−0.492 ~ 2.607

IQR: Interquartile Range.

Low/Middle/High (%): Low refers to the percentage of the population below the 25th percentile, middle refers to the IQR, and High refers to the 75th percentile and above.

† The Japanese version is a 5-point Likert scale including intermediate item 3.

## 4. Comprehensive discussion

### 4.1. Proportion of underweight women and their birth weight

First, the screening survey’s results revealed that the average BMI for underweight groups was 17.41 kg/m^2^, and the percentage of the underweight group was 23.54% of the total number of participants. This high percentage is comparable to that reported previously (4, 5). Concerning weight fluctuations among underweight women, the percentage of respondents who responded “Easy to lose” was higher than that of normal-weight women. Furthermore, among underweight participants, this percentage was higher for those who had no experience with dieting. These results suggest that the underweight participants in this study, who had never been on a diet, were potentially thin. The underweight group and NDG were more likely to respond positively to questions regarding eating habits, such as *want to gain more weight* and *want to increase food intake*. Although this study did not examine the desire to lose weight, the results suggest that women who are underweight and have NDG, are less likely to have the desire to lose weight.

No differences were found between the underweight and normal-birth-weight groups. Meanwhile, a comparison of underweight according to dieting experience revealed that the underweight group was significantly less likely to have ever been on a diet. The underweight group also had the highest percentage of LBWs; according to data on LBWs in Asia/Pacific reported in 2020 (26), Japan has 9.5% thereof, a situation that is higher than any other developed country (27). In the present study, the prevalence of underweight women was 10.6%, which was approximately 1% higher than that reported in previous studies. Preconception and early pregnancy nutritional status confer a risk for adverse pregnancy outcomes, such as LBW. These are explained not only by factors such as poor weight gain owing to limited caloric intake in thin women (28), but also by a conceptual framework of pregnancy stages that may be affected by nutrition (29). It has also been demonstrated that singletons born to underweight women are at a higher risk of having LBW than those born to normal-weight women (30). Furthermore, women born underweight have a 3.6-fold increased risk of gestational diabetes when they become pregnant (31). Newborns who are undernourished *in utero* are at risk of developing diabetes and lifestyle-related diseases in the future, affecting them during the perinatal period, early childhood, and adulthood (32). In this study, we did not investigate the influence of the participant’s mother’s body mass, nor did we inquire regarding the birth weight of the participants’ children, if any. Therefore, further examining the background leading to being underweight—by investigating both sides of this issue—is necessary.

### 4.2. Perception of body mass index (BMI)

Previous studies have reported that not only women having normal weight but also underweight women perceive themselves as overweight, and these are found, for example, in adolescents who desire to be thin (6, 33). Reportedly, in Japan, young women who are underweight do not perceive themselves as thin compared with other age groups (34). It is possible that having a distorted body image may lead to an unnecessary desire to lose weight, or a contradictory desire to lose more weight, while recognizing that their current body shape is appropriate. However, in this study, approximately 65% of underweight women were identified as skinny, and less than 8% were identified as obese. Negative body image decreases with age (35), and middle-aged and older generations focus more on body function and ability than on external criteria. These factors may have influenced the age range of the participants in this study, who were between 18 and 29 years old. Underweight individuals who had no experience with dieting, were slightly less likely to respond to obesity than those who had been on a diet. Most underweight participants were considered to have no significant gap between their body image and actual BMI. The BMI and current weight of underweight participants were generally consistent, and nearly 80% of the respondents answered “Normal/Satisfied” in terms of body shape satisfaction.

As indicated by BMI, body size is associated with greater body dissatisfaction among adult women (36). In the present study, compared to underweight individuals, normal-weight individuals were significantly more likely to respond as being obese (45%), and underweight individuals were also less likely than normal-weight individuals to have ever dieted. Additionally, approximately 43% of normal-weight respondents also reported dissatisfaction with body shape, which supports the study by Weinberger et al. (36). Irrespective of whether they have ever dieted, they may be underweight and view their current weight (BMI) as at least an ideal body shape. By contrast, approximately 44% of the group with dieting experience provided the answer that weight was easy to gain, significantly more often than the group without dieting experience, especially for weight fluctuations, and approximately 67% answered “disagree” for wanting to gain more weight significantly more often than the group without dieting experience. Unacceptable and obese weights were also less common in the DG. The maximum weight was reported to be significantly less by the NDG, which was considered constitutionally thin. The fact that the ideal weight reported by NDG was higher than their current weight also supports the idea that they have little resistance to weight gain. However, it can be inferred that women who are underweight and in the DG are resistant to gaining weight, assuming that their current body shape is ideal. Nevertheless, the weight (BMI) that underweight women perceived as unacceptable/obese corresponded to normal weight, despite their previous dieting experience. It is possible that underweight women, whose previous maximum weight corresponded to a BMI of normal weight, were dissatisfied with their body shape at that time; capturing more detailed information on these issues in the future is necessary. Previous studies have suggested that women with higher BMI tend to have lower body satisfaction (36, 37). Therefore, BMI can be used to predict body satisfaction (38). Considering the existence of a proper weight (BMI) barometer for oneself as perceived by the underweight group, considering an educational approach that considers BMI and body satisfaction along with dieting experiences in health awareness is necessary.

### 4.3. Exercise habits

Motivations for exercise participation vary and include improving or maintaining health and fitness, gaining enjoyment from exercise, and physical and psychological benefits. Other motivations include weight loss or maintenance, improvement of muscle strength or appearance, and self-presentation purposes such as developing a healthy image (39). Japan aims to increase the percentage of adults (aged 18–79) who exercise at least once a week, from the current 56 to 65% to prevent and improve lifestyle-related diseases and extend healthy life expectancy through care prevention (40). In the past year, the participants in this study were classified as underweight, having normal weight, and obese, each of which exercised at least once a week. There were no significant differences in past exercise habits (elementary school to high school age), with a decrease in each implementation rate as the age group increased. From the summary of recommended levels of physical activity, published by the WHO (41), the recommended duration of exercise for adults is 150–300 min/week. Considering these findings, the participants of this study did not meet these criteria. In Japan, it may be possible to promote exercise habits by considering activities in school physical education.

However, an evident difference was found when focusing on being underweight and DG. In all but high school age groups, the percentages of underweight and NDG were significantly lower than those with exercise habits. The proportion of adult Japanese women who exercised at least once a week during 2018–2021, ranged from 51 to 54% (42). Among women who are underweight and DG, 60% have an exercise habit that is close to the goal of Japan’s Basic Plan for Sports. However, the percentage of NDG was low (40%). The average number of exercise hours was approximately 1 h, regardless of the dieting experience. However, the average number of exercise days (per week) was about 3 days for DG and 1 day more than average. Around 87–90% of the non-diet experience group answered “Neutral/Agree” regarding the importance of current and future exercise practices. However, only a small percentage (19%) were willing to act thereon. Those who lost weight were more likely to perform an exercise routine. To date, no studies have elucidated why women who are underweight and have never engaged in dieting behaviors take no action, despite the perception that exercise is important. Two interesting findings of this study were that underweight diet-naïve women were less resistant to gaining weight and had fewer exercise habits. There is a need to elucidate the factors that prevent underweight women without slimming behaviors (i.e., constitutionally thin) from having adequate exercise habits. However, at least one combination of these two perspectives should be examined.

There was no specific difference between the underweight and normal-weight groups for the item reasons for having an exercise routine. By contrast, the comparison to dieting experience revealed that the proportion of respondents with DG was significantly higher in three categories, namely “To feel inadequate in physical exercise,” “For spiritual cultivation or training,” and “Beauty and obesity reduction.” The results indicated that the proportion of respondents with a DG was significantly higher. Notably, 70% of the DG answered “Beauty and obesity reduction,” while the NDG was 27.2% less likely to answer this question (44%). Previous studies have found a relationship between BMI, exercise frequency, and motivation to exercise (43). Women with a higher BMI tended to be more motivated to exercise for weight loss, and those with a higher exercise frequency tended to be motivated by positive aspects, such as health. In the present study, the underweight and DGs were resistant to weight gain. The weight they considered unacceptable/obese (BMI) was lighter than that of the NDG. Therefore, even among women with low BMI, exercise motivation may be enhanced by having a dieting experience and desire to lose weight, which needs to be verified.

Body image is a multidimensional construct that reflects thoughts, emotional evaluations, and perceptions of one’s body (44). It can be categorized into states and characteristics; consistent findings have been reported that exercise positively affects it (45). Physical activity and participation in sports are associated with positive body image (46); thus, participation in such activities may eliminate negative body image, which is considered a form of weight and shape dissatisfaction. As such, it contributes to increased participation in physical activity and sports. Greater effects are expected for women since they are more likely than men to report body image disturbances (45). For these reasons, exercise is considered a promising option to improve women’s body image dissatisfaction. This study’s results suggest that the question is how to improve the rate of exercise among underweight women, especially those who have never previously dieted and are not considered to have a desire to lose weight. There may be factors, such as the image of exercise, or weight loss, does not match their body image. It is necessary to raise awareness of the need to motivate people to exercise and create a positive perception of the need to continue exercising. Additionally, approaches to underweight women who do not have exercise habits, include providing appropriate opportunities for exercise and overcoming their dislike for it. Moreover, it is necessary to provide good examples of how to secure the time allocated for exercise and opportunities to learn programs that women can practice. From a long-term preventive perspective, raising awareness of the need for exercise habits in childhood is necessary, and these efforts must also play a role in the educational field.

#### 4.3.1. Eating habits

In this study, there were no significant differences in perceptions of the importance of eating habits or the frequency of missed meals, when comparing underweight to normal-weight groups and underweight to diet experiences, respectively. Moreover, there were no serious problems with the diet or nutritional intake. Concerning the importance of current and correct eating habits, the results showed a higher percentage of underweight than normal-weight individuals. Underweight women, who desire to lose weight, tend to pay more attention to improving their perception of their body composition and eating habits (8), and the results support these findings. A study examining the frequency (in days) of major meal intake among underweight, normal-weight, and obese young women reported no differences in the frequency of missed meals. Underweight women exhibited the highest frequency of snacking (70%) (47). The present study’s results are consistent with those of previous studies concerning the frequency of missed meals. This study did not ask specific questions to determine health status, such as the number of meals and snacks consumed, the time of day they were consumed, preferences, and sleep status; therefore, a comprehensive survey of these factors is needed.

One noteworthy finding of this study was that underweight patients with NDG were less aggressive in taking specific actions to maintain proper eating habits. Contrastingly, the DG was more aggressive. A similar trend was observed in the question on exercise habits, but a lack of exercise habits and eating a small diet may lead to health risks. For example, weight loss lowers basal metabolism and the amount of energy expended. In this case, relative muscle mass may be reduced, and there is an increased risk of type 2 diabetes, similar to obesity, even if one is thin (1, 48, 49). As regular exercise activates autonomic activity related to energy metabolism, eating more food for this purpose does not increase body weight. Health benefits in terms of weight regulation and heat production were significant. By contrast, approximately 50% of the diet experienced group was reluctant to increase the amount of food they ate, with approximately 50% stating that they “disagreed” with increasing food intake. Additionally, a higher percentage of the DG reported that stress caused them to overeat. However, the NDG reported an appetite decrease of approximately 35%. There is a psychological background to changes in food intake in response to stress. Women who are stress inhibited over-eat during specific or general stress experiences (49). Women who are underweight and engage in behaviors aimed at slimming down also need to focus on emotional regulation issues, as they may engage in overeating behaviors under stress (50).

Young women who are normal-weight or underweight may not feel the need to change their lifestyle, and a prolonged period of unhealthy lifestyle habits can harm their physique and health status. As future health status is related to current behaviors, it is important to promote healthier lifestyles rather than focusing solely on maintaining an appropriate physique and weight. Nutritional intake has become more diverse in this age of fads and zeitgeists; for example, supplementation is used to supplement deficient nutrients. Considering these trends, health education, including nutritional supplements, is a public health issue that must be addressed to achieve a healthy diet. Adopting an approach to educate young people to practice healthy lifestyle habits for preventing future lifestyle-related diseases is important.

### 4.4. Comparison of questionnaires

#### 4.4.1. Eating attitude questionnaire (Eat-26)

The study revealed that the underweight and DG had an average EAT score of 10.70 ± 12.75 points, which qualified as a midrange eating disorder (≥ 9 points). Moreover, in the DG, the percentage of women with a severe eating disorder (≥ 20 points) was approximately 2.3 times higher (15.5%) than that in the NDG (6.7%). In other words, it is difficult to overlook that one of every six underweight DG participants had severe eating disorders. Underweight young women with a desire to be thin have been reported to have higher EAT scores than similar normal-weight women (51). The desire to lose weight may increase the risk of developing eating disorders (52). In particular, the desire to be thin and the risk of developing eating disorders have been reported in adolescents (53, 54).

This study’s results revealed that the age at which the underweight DG became concerned about their weight gain and decided to lose weight was between 17 and 18 years. Thus, the awareness of weight loss may increase at this age. Women who are underweight, desire to lose weight, and have dieted are associated with eating disorders (51). In cases of being underweight and having a history of dieting, the potential impact on future health should be addressed. Given these considerations, there is a need to promote appropriate health education for this population, along with examining other mediating factors.

#### 4.4.2. eHealth literacy

Effective use of health information on the Internet requires the ability to search and find such information appropriately, understand and evaluate it, and use the knowledge gained to address and solve health problems (13). This is defined as eHealth literacy (13). This study revealed no difference in eHEALTH scores between the normal weight and underweight groups. However, the underweight DG scored significantly higher than the NDG did. The eHEALTH high group (75th percentile or higher) was approximately 10% higher than that of the NDG. It is possible that underweight and dieting young women actively use the website to gather information about effective diets. Therefore, a higher eHEALTH score for this group was expected, and the hypothesis was supported. Since 2020, the world has been affected by the coronavirus disease (COVID-19). This has led to the recommendation of improved digital healthy diet literacy and eHEALS. For example, people may be able to maintain eating behaviors, physical activity, and mental health and protect their mental health (55). Previous studies have also demonstrated that individuals with high eHealth literacy are significantly more likely than those with low eHealth literacy to exhibit healthy behaviors, such as physical exercise and a balanced diet (22). In this study, 32.8% of the underweight and diet-naïve groups were in the low- eHEALTH group (< 25th percentile). There is a need to educate underweight and low-eHEALTH literacy groups to increase their eHealth. Simultaneously, it is necessary to further investigate the relationship between eHealth and health behaviors in the group with high eHealth literacy and dieting experience, such as whether it is linked to excessive dieting behavior.

#### 4.4.3. Sociocultural attitudes toward appearance (SATAQ-3 JS)

Women who desire to lose weight are more likely to internalize information from the media and experience subjective cognitive effects in the form of body image distortions. Internalization of information from the media may be highly associated with body dissatisfaction, the desire to lose weight, and the tendency toward dieting. Tis study’s results support these hypotheses, as underweight women who have dieted may be particularly susceptible to sociocultural standards of beauty portrayed in the media. In particular, the DG scored higher on the Internalization General Scale, indicating a strong internalization of the portrayed ideal. Women with a higher BMI reported feeling more sociocultural pressure from parents, peers, significant others, and the media to have a culturally acceptable body. They reported feeling more sociocultural pressure (43). Anxiety experienced by individuals who perceive their body shape to be negatively evaluated by others is defined as social physique anxiety (56). Individuals with prominent levels of social physique anxiety are more likely to have improved appearance. It has been suggested that people with high social physique anxiety exercise to improve their appearance and decrease such anxiety. The underweight and DG groups in this study may fall into this category. They may have exercise habits owing to pressure to reach an ideal body, or to look more attractive, and measures to soften them are needed. New efforts are needed to focus on groups that internalize media messages. Sustained awareness raising should be conducted based on media literacy and psychological aspects to prevent inactivity in food and exercise behavior, such as body image distortion, slimming desires, excessive dieting behavior, and body dissatisfaction.

#### 4.4.4. Personality characteristics (TIPI-J)

Previous studies have examined the relationship between personality traits and health behaviors across various age groups. For example, it has been found that higher health literacy is associated with higher openness and diligence in older adult individuals (57). Additionally, higher openness and industriousness tend to be associated with healthier eating behaviors (58, 59). This elevated level of openness may lead to a high interest in health behaviors and education, as people are interested in various subjects and exhibit curiosity and fantasies. The opposite was true in the present study, with the NDG scoring higher in openness than the DG. In the case of the dieting experience, participants may engage in health behaviors to lose weight based on the desire to lose weight or the need to lose weight. However, in the case of NDGs, even if they desire to lose weight owing to high openness, they may remain pessimistic, fantasize, and may not take actual health actions.

The percentage of respondents who answered “Agree” to the question “Going to take specific actions for exercise habits” was significantly lower than that of the DG (19%). The same trend was observed for eating habits, with a significantly lower percentage of respondents agreeing with the “Will take action to develop good eating habits” question than those in the DG. Therefore, even if respondents recognized the importance of these habits, they did not act to develop them. For example, a high percentage of respondents with dieting experience answered “Beauty and obesity reduction” as reasons for exercising. Thus, one of the factors contributing to the inability to act was the lack of sufficient purpose and goal thinking. Regarding diligence, those who had dieted scored higher than those who had not. This result supports previous studies (58, 59) and may indicate that the diet was implemented owing to high health literacy and efforts to lose weight. A high level of diligence indicates that individuals work hard to achieve their goals and take things seriously, with a strong sense of responsibility. It has also been demonstrated that women are more likely to diet (controlling their food intake) when they are dissatisfied with their body shape or weight. Therefore, the results suggest that those with dietary experience were more diligent since they wanted to lose weight.

#### 4.4.5. Self-esteem (RSES)

Exposure to media images of slim and beautiful women has been reported to negatively affect young women’s body image and moods (60). The reason for this is believed to be the internalization of the thinness ideal, in which daily exposure to messages that idealize thinness causes women to adopt it as their values and ideals (61). Body image and moods were normal when the thin ideal was low. However, increased internalization of the slender ideal is, reportedly, a risk factor for increased self-image dissatisfaction and development of abnormal eating behaviors (61). Adolescents and young adults internalize physically beautiful body shapes. If internalization is unsuccessful, dissatisfaction with their bodies and the constant search for it may result in risky behaviors, that may lead to the development of eating disorders (62). Even if the development of a mental disorder, such as an eating disorder, does not occur, the strength of the ideal of thinness is significantly positively correlated with depressive tendencies, anxiety, and low self-esteem (63). Therefore, it is conceivable that under the influence of the media and other factors, people try compensating for their undesirable self-image and increase their self-esteem by becoming a body type that fits fashionable trends.

As the evaluation of self and body shape are related, it is conceivable that those with a sense of inadequacy toward the self and a vague sense of insecurity and emptiness in their daily lives, are more likely to perceive disadvantages in their current body shape. However, there were no significant differences in neuroticism or self-esteem in the personality traits among the participants in this study. Therefore, there may be no difference in whether a person has ever dieted, unless the eating disorder is severe, the person has morbid anxiety, or a body complex. Indeed, higher self-esteem has been associated with fewer worries and anxieties about body image and with the promotion of positive evaluations of one’s weight and shape (19). This study’s results indicate that the average score for both underweight and normal-weight groups was around 29, which suggests that the respondents did not have an overly negative evaluation of their body shape and did not have an overly slim ideal. Self-esteem is the emotional evaluation of an individual, such as how one evaluates oneself, and is also considered an important predictor of implementing health behaviors. This may be related to one’s appearance, positive attitudes, and communication skills. For example, high self-esteem has been demonstrated to positively affects sports participation (64). Thus, high self-esteem positively affects an individual. Participants in this study, both normal-weight and underweight, exhibited approximately 20–25% of their total self-esteem scores below the 25th percentile. As low self-esteem may result in a low self-evaluation, focusing on the group with low self-esteem and further investigating the relationship with health behaviors, such as exercise and eating habits, is necessary to capture their characteristics.

## 5. Limitations of this study

This study focused on the dieting experiences of underweight young women and collected basic health habits data, such as weight perceptions, past and current exercise habits, and eating behavior habits. In addition, psychological variables such as media influence on body image, e-health literacy (such as the ability to appropriately search for and utilize health information on the internet), personality traits, and self-esteem were used to identify and comprehensively examine multifaceted influences carefully. The strength of this study is that the data acquired will contribute to the development of applied research. However, this study has three limitations. First, as this was a cross-sectional study, future research should consider follow-up investigation to corroborate its results. Second, this study was limited to analysis for background validation purposes and did not address associations among the questionnaires. Future studies should analyze these data as an application area to explore their relevance. Finally, when recruiting participants for this study, it was assumed that they would be able to report their birth weight recorded in their MCH Handbook, which reduced the number of responses to this survey, especially in the underweight group. There is scope for further study regarding whether there is a difference between participants who can report their birth weight based on the MCH Handbook and those who cannot. The difference in the percentage of responses to some of the questions may be partially due to this effect. Therefore, a wider target group should be considered to test the external validity of this study’s results.

## 6. Conclusion

This study examined the background of underweight young women in Japan, focusing on their dieting experiences. The results revealed that the two groups had divergent backgrounds. A high percentage (23.54%) of participants were underweight. Approximately 65% of the underweight subjects identified themselves as *Skinny* and 8% as *Obese*, suggesting no significant gap between their actual body shape and their perceptions. Although there was no difference in current weight (BMI) based on dieting experience, underweight DG’s had difficulty losing weight, tended to overeat during times of stress, and were resistant to increasing food intake and weight gain. The percentage of respondents in the DG who exercised, from past to present, was significantly higher than those in the NDG, and 70% reported beauty and obesity reduction as the main reasons for their exercise habits, which was significantly higher than those in the NDG. This response may have increased owing to the impact of exercise on body image, as a form of body satisfaction. Conversely, the NDG declared their ideal weight to be slightly heavier than their current weight. Although they were more likely to lose weight and responded positively to gaining weight and eating more, a greater percentage of respondents reported reduced appetite during stress. The birth weight was significantly lower for the NDG group, suggesting a potential tendency to be underweight. The percentage of respondents who exercised, from elementary school to the present, was less than 40%, and a high percentage of respondents tended to dislike exercise or had never had the opportunity to do so. The importance of exercise and eating habits was positively recognized regardless of the dieting experience, but those who had never dieted before could not take actual action.

Standardized questionnaires revealed no difference in self-esteem by dieting experience, with roughly one in four underweight women having low self-esteem. DG was associated with EAT scores in the midrange eating disorder range, and eHealth Literacy, SATAQ, and the conscientiousness (TIPI-J) scored significantly higher than the NDG. This result raises concerns that those who are underweight and have dieted may engage in behaviors that lead to stoicism, thinness, and progress to eating disorders. The tendency to internalize media information also suggests a need for health education specific to this population, since the possible effects on subjective cognition may occur in the form of body image distortion. The NDG had significantly higher openness (TIPI-J) than the DG, suggesting that they were more open to experience. Openness is a trait that favors new experiences and practices. Therefore, they are expected to be more positive toward health behaviors. However, their exercise and eating habits tended to be less active when we examined them, confirming a gap between personality traits and behavioral aspects. Young, thin women who eat little and do not exercise are at risk of future health risks. Therefore, it is suggested that they be educated to obtain appropriate exercise and eating habits. As described above, the results suggest the need for different health education programs for thin women who desire to lose weight and experience dieting, compared to those who do not. This study’s results can be used to examine sports opportunities optimized for each individual and measures to ensure adequate nutritional intake. These efforts will help women lead long, healthy, and fulfilling lives. We hope that this study’s results will be widely used to promote future research in underweight women.

## Data availability statement

The raw data supporting the conclusions of this article will be made available by the authors, without undue reservation.

## Ethics statement

The studies involving human participants were reviewed and approved by Research Society Ethics Committee of the Faculty of Health and Sports Science and the Graduate School of Health and Sports Science, Juntendo University, Japan. The patients/participants provided their written informed consent to participate in this study.

## Author contributions

YM (Sports Medicine and Sports Psychology) and YT (Sports Medicine and Sportology) designed this study, collected all data, performed the statistical analysis, and prepared the manuscript. SY (Health Psychology) assisted in the development of the questionnaire and assisted with the analysis and coordination of the manuscript. YY (Healthy Life Expectancy) assisted with the analysis and provided comments according to specialty. HK (Athletic Training, Kinesiology) assisted with the design of the paper and coordination of the manuscript and provided comments according to their specialty. HO (Sports Medicine and Sportology), KO (Metabolism and Endocrinology), and HK (Metabolism and Endocrinology) assisted in the development of the questionnaire and coordination of the manuscript and provided comments according to their specialty. All authors contributed to the article and approved the submitted version.

## Funding

This study was supported by the Private University Research Branding Project of the Japanese Ministry of Education, Culture, Sports, Science, and Technology. It was also supported by research funding from the Institute of Health and Sports Science and Medicine, Juntendo University; and the Joint Research Program of Juntendo University, Faculty of Health and Sports Science, JKA through its promotion funds from AUTORACE.

## Conflict of interest

The authors declare that the research was conducted in the absence of any commercial or financial relationships that could be construed as potential conflicts of interest.

## Publisher’s note

All claims expressed in this article are solely those of the authors and do not necessarily represent those of their affiliated organizations, or those of the publisher, the editors and the reviewers. Any product that may be evaluated in this article, or claim that may be made by its manufacturer, is not guaranteed or endorsed by the publisher.
