# Supplementary material for: Multidimensional background examination of young underweight Japanese women: focusing on their dieting experiences
Source: Front Public Health. 2023 Jun 2;11:1130252. doi: 10.3389/fpubh.2023.1130252 (PMC10273403; doi:10.3389/fpubh.2023.1130252)
Supplement: Supplementary file 1 [file Table_1.docx]

**Supplementary Table 1.** **Questionnaire List**

| **(a) Participant demographic data questions** | | **Answer** |
| --- | --- | --- |
| Age | | years |
| Age of the first menstruation | |  |
| Height | | cm |
| Weight | | kg |
| BMI | | Kg/m^2^ |
| Birth weight‡ | | g |
| Maximum weight | | Kg/m^2^ |
| Ideal weight | |  |
| Weight perceived as unacceptable | |  |
| Weight in kg perceived as model shape | |  |
| Weight perceived as obese | |  |
| Age at which weight gain became a concern§ ^[M]^ | | Years |
| Age | |  |
| Annual income *　　　　　　≦2,000,000 yen | | n / % |
| 2,000,000 yen～＞4,000,000 yen | |  |
| 4,000,000 yen～＞6,000,000 yen | |  |
| 6,000,000 yen～＞8,000,000 yen | |  |
| 6,000,000 yen～＞8,000,000 yen | |  |
| 10,000,000 yen≧ | |  |
| I do not know | |  |
| I prefer not to answer | |  |
| **(b) Diet experience, body image, weight fluctuation, gaining weight†** | | **Answer / Choices** |
| b1. Diet experience ^[M]^ | | Yes / No |
| b2. Body image | | Skinny/ Normal /Obese |
| b3. Body shape satisfaction | | Dissatisfied / Normal / Satisfied |
| b4. Weight fluctuation ^[M]^ | | Easy to lose / Unchanged / Easy to gain |
| b5. Want to gain more weight | | Disagree / Neutral / Agree |
| **(c) Exercise habits questions** | | **Choices** |
| 1-1. Exercise habits in the past year | | Yes / No |
| 1-2. Elementary school age | |  |
| 1-3. Junior high school age (club activities) | |  |
| 1-4. High school age (club activities) | | Yes / No / Not applicable‡ |
| 2. Sports to continue throughout your life | | Individual / Group / None |
| 3. Current exercise habits are important | | Disagree / Neutral / Agree |
| 4. Future exercise habits are important | |  |
| 5. Going to have an exercise routine in future | |  |
| 6.Going to take specific actions for exercise habits | |  |
| **(c-1) Reasons for exercise habits** | **(c-2) Reasons for not having exercise habits** | **Choices (multiple answers)** |
| c1-1. Health and fitness | c2-1. Too busy | Yes / No |
| c1-2. Fun or Distractions | c2-2. Physically weak |  |
| c1-3. To feel inadequate in physical exercise | c2-3. Old age |  |
| c1-4. For spiritual cultivation or training | c2-4. No place or facilities |  |
| c1-5. To improve my record or ability | c2-5. Do not have friends |  |
| c1-6. To contact family | c2-6. Do not have a mentor |  |
| c1-7. Socializing with friends and colleagues | c2-7. Costs money |  |
| c1-8. Beauty and obesity reduction | c2-8. Do not like exercise/sports |  |
| c1-9. Club activities | c2-9. Never had the chance |  |
| c1-10. To relieve stress | c2-1. Too busy |  |
| **(d) Eating habits questions** | | **Choices** |
| d1. Current food intake is good ^[M]^ | | Good / Neutral / Problematic |
| d2. I get the nutrients needed from my daily diet ^[M]^ | | Adequate / Sufficient / Insufficient |
| d3. Current eating habits are important | | Disagree / Neutral / Agree |
| d4. Future eating habits are important | |  |
| d5. Want to have the right eating habits | |  |
| d6. Will take action to develop good eating habits | |  |
| d7. Want to increase food intake | |  |
| d8. Frequency of missing meals ^[M]^ | | Every day / Several time a week / None at all |
| d9. Stress and fatigue appetite change ^[M]^ | | Decrease / No change / Over-eat |

*Note*. *: Annual income: Salaried full-time employees who had an average salary of 4,430000 yen (men: 5,450,000 yen; women: 3,020,000 yen) (24).

[M]: Questions for the main survey.

‡ Only responses recorded in the Maternal and Child Health Handbook were collected.

§ Data from respondents concerned about weight gain.

‖: Data from respondents with diet experience.

¶: Not applicable are participants who have not been enrolled in high school.
